# Factors influencing global antiretroviral procurement prices

**DOI:** 10.1186/1471-2458-9-S1-S6

**Published:** 2009-11-18

**Authors:** Veronika J Wirtz, Steven Forsythe, Atanacio Valencia-Mendoza, Sergio Bautista-Arredondo

**Affiliations:** 1 Center for Health System Research, National Institute of Public Health, Mexico; 2 Futures Institute, Glastonbury, Connecticut, USA; 3 Center of Evaluation Research and Surveys, National Institute of Public Health, Mexico

## Abstract

**Background:**

Antiretroviral medicines (ARVs) are one of the most costly parts of HIV/AIDS treatment. Many countries are struggling to provide universal access to ARVs for all people living with HIV and AIDS. Although substantial price reductions of ARVs have occurred, especially between 2002 and 2008, achieving sustainable access for the next several decades remains a major challenge for most low- and middle-income countries. The objectives of the present study were twofold: first, to analyze global ARV prices between 2005 and 2008 and associated factors, particularly procurement methods and key donor policies on ARV procurement efficiency; second, to discuss the options of procurement processes and policies that should be considered when implementing or reforming access to ARV programs.

**Methods:**

An ARV-medicines price-analysis was carried out using the Global Price Reporting Mechanism from the World Health Organization. For a selection of 12 ARVs, global median prices and price variation were calculated. Linear regression models for each ARV were used to identify factors that were associated with lower procurement prices. Logistic regression models were used to identify the characteristics of those countries which procure below the highest and lowest direct manufactured costs.

**Results:**

Three key factors appear to have an influence on a country's ARV prices: (a) whether the product is generic or not; (b) the socioeconomic status of the country; (c) whether the country is a member of the Clinton HIV/AIDS Initiative. Factors which did not influence procurement below the highest direct manufactured costs were HIV prevalence, procurement volume, whether the country belongs to the least developed countries or a focus country of the United States President's Emergency Plan For AIDS Relief.

**Conclusion:**

One of the principal mechanisms that can help to lower prices for ARV over the next several decades is increasing procurement efficiency. Benchmarking prices could be one useful tool to achieve this.

## Introduction

### Why do antiretroviral medicines (ARV) prices matter?

Since the mid 1990s, highly active antiretroviral treatment (HAART) has become the standard recommended treatment, including a combination of at least three antiretroviral medicines. Between 2002 and late 2007, the number of patients receiving antiretroviral treatment (ART) worldwide grew from around 300,000 to approximately 3 million people [1].

However, there remains a large gap between those in need of receiving HAART and those who receive it. According to estimates, out of the 33.5 million people living with HIV/AIDS, 10 million need HAART, which leaves 7 million people living with HIV and AIDS (PLWHA) currently untreated. This is particularly problematic in Sub-Saharan Africa, where two-thirds of all PLWHAs are residing [2]. Furthermore, it is estimated that 13.7 million people in 2010 and 21.9 million people in 2015 will need ART [2].

Affordability remains a critical issue, despite the fact that between 2000 and 2007, the median price for first-line combination therapy in developing countries fell from US$10,000 to about US$90 per patient per year [3]. But even US$90 remains unaffordable for many low-income countries to spend on care for PLWHA, even when considering the growing availability of donor funds. In addition, an increasing number of PLWHA require second-line treatment because of resistance to first-line drug treatment or an inability to tolerate first-line drugs. As a result, many low- and middle-income countries are struggling to provide sustainable access to HAART.

In 2007, the WHO reported that the median price for the most frequently used second-line HAART (abacavir + didanosine + lopinavir/ritonavir) for low-income countries was US$1,214, 13.5 times higher than for first-line treatment. In middle-income countries, the price for second line therapy was 36.3 times higher than for first line therapy (US$3,306 for second-line therapy, as compared to US$91 for first-line therapy) [4]. Similarly, Médecins sans Frontières (MSF) reported that, according to manufacturer price information, a change from the cheapest first-line regime quoted with US$87 to the cheapest second line with $US749 (tenofovir + emtricitabine + lopinavir/ritonavir) will at least increase expenditure nine-fold [3]. As PLWHA on HAART live longer, an increasing number will require second- and third-line therapies. The durability of first-line therapy greatly varies between regions; overall, it has been estimated that 22% of patients switch after a mean time of 20 months to a second-line combination [5]. An analysis of factors influencing ARV prices is very relevant to increase long-term efficiency (best value for money) in the provision of HAART. This would allow designing appropriate policies to fuel the production of low price ARVs or to implement the most effective procurement processes.

### One world, one price?

A first hypothesis that could be tested is that antiretroviral prices do not differ significantly across countries. In fact, some pharmaceutical companies, such as Pfizer, have introduced new medications (e.g. maraviroc) at one global price, without any tiered pricing or discounts provided to poorer or countries with higher prevalence [3]. Such an approach has numerous advantages, including eliminating the risk for arbitrage (purchasing medications in a low price country and reselling them in a higher priced country) [6] and limiting objections from some countries about why they are paying more than others. It may also be argued that having one global price is more equitable since every country pays the same price (although alternatively it may be argued that such a pricing policy is inequitable because it does not recognize the lower ability of poorer countries to pay).

The pricing data on ARVs, however, overwhelmingly disproves the hypothesis that prices do not vary from country to country. For example, the average price per patient per year paid for by lower income countries for lopinavir/ritonavir 133/33 mg is US$500, whereas the price for the same product in middle-income countries is US$1134 [4]. Pharmaceutical companies have tried, in some cases, to rationalize these price differences by establishing tiered pricing based on socioeconomic status and/or other factors (Merck, 2008) [7]. Even with these attempts, however, countries have nevertheless been able to negotiate significant discounts on ARVs which are not available to other neighboring countries [3].

Other than the socioeconomic status of a country, the factors potentially responsible for the observed differences in prices are numerous, including the prevalence of HIV, the volume, the role of third party negotiators such as the Clinton HIV/AIDS Initiative [8] or the United States President's Emergency Plan For AIDS Relief (PEPFAR) [9], and access to generics. Vasan et al [10] found that differential prices are inconsistently applied, particularly among lower middle-income countries which are charged higher prices. Similarly to Vasan et al [10], Chien [11] used the Global Price Reporting Mechanism (GPRM) database to analyze both volume and ARV prices in sub-Saharan Africa, concluding that, despite differential pricing, generic drugs were still being purchased at significantly lower prices than innovator products.

The objectives of the present study were twofold: first, to analyze global ARV prices between 2005 and 2008 and associated factors, particularly procurement methods and key donor policies on ARV procurement efficiency; and, second, to discuss the options of procurement processes and policies that should be considered when implementing or reforming access to ARV programs. Given the limited analysis of the impact of procurement methods and policies on procurement prices, the present study could provide important policy recommendations for individual countries and also for donor organizations. Should all countries or HIV/AIDS programs be recommended to use a third party negotiation strategy to achieve lower prices? Should countries or HIV and AIDS programs always choose generic ARV over innovator products if patent policies allow doing so? Does bulk procurement result in lower prices? 

## Methods and data sources

In order to analyze factors influencing global ARV prices, the Global Price Reporting Mechanism (GPRM) was analyzed. While other sources use price quotes from manufacturers [3], the strength of the GPRM is that it provides information on the ARV prices that countries actually paid (note: not end users of the drugs). The majority of the information is transactional data for ARV procurements made with donor funds from the Global Fund for AIDS, Tuberculosis and Malaria (GFATM). Other data come from the country offices that report procurement prices to the World Health Organization (WHO), as well as international organization and procurement agencies, such as Mission Pharm, United Nations Children's Fund (UNICEF), International Dispensary Association Foundation (IDA). These prices are all posted by the WHO on their publicly accessible database http://www.who.int/hiv/amds/price/hdd/. For this study, information was downloaded in March 2009. We used procurement data from January 2005 to December 2008, reported for twelve of the most frequently used adult ARV medicines in first-line and second-line therapy regimes in developing countries: efavirenz 600 mg, lamivudine 150 mg, lamivudine 150 mg/zidovudine 300 mg, nevirapine 200 mg, stadivudine 40 mg and zidovudine 300 mg as first-line therapy; and abacavir 300 mg, didanosine 100 mg and 400 mg, lopinavir/ritonavir 133/33 mg, ritonavir 100 mg and tenofovir 300 mg as second-line therapy. (We used the WHO classification of first- and second-line therapy [4]).

A total of 11,276 transactions were available for an analysis the 12 chosen ARVs (minimum of 332 transactions for ritonavir to 1,732 for nevirapine) (Additional file 1, Table 1). In total, 92 countries reported data to the GPRM, 48 of them classified as low-income countries, the rest lower-middle, upper-middle and very few high-income countries. Out of all transactions, 84.6% were made by countries which participated in the Clinton HIV/AIDS Initiative (CHAI) in 2008 and 40% of purchases were made by PEPFAR focus countries. All prices in the present study are reported in US dollars, unadjusted for inflation. This is to allow comparison with other international literature on drug prices that also use prices unadjusted for inflation [3,4]. In some cases, very high prices were corrected when it was obvious that this was due to a recording error; for instance, when instead of unit price the package price was reported (a multiplication of the number of units per package by unit price). Also donations were eliminated (purchases reported with a price of US$0).

First, for each of the ARV and year of study, the median price per patient per year was calculated. Since prices were not normally distributed, the prices at the 25th and 75th percentiles of the sample are also reported.

In order to identify the principal factors influencing prices of each ARV, linear regression models were used choosing the following independent variables based on their theoretical importance on price: HIV country prevalence (<2%, 2-5%, >5%) [1], volume (in terciles), national income per capita using the World Bank classification (low-income, lower-middle-income, upper-middle-income) [12], AIDS Program Effort Index (API) [13], whether the product procured was innovator or generic product (determined by the database entry of the manufacturer which sold the ARV), whether the country is member of CHAI and whether the country is one of the 15 focus countries for the United States President's Emergency Plan For AIDS Relief (PEPFAR) [14]. (PEPFAR operates in 114 different countries, but only 15 countries are identifies as "focus countries".) High-income countries were excluded from the regression models since it is likely that the factors influencing prices in those countries are different than in other income groups. In addition, the number of procurements reported was very small. 

Prices were transformed in the logarithm as they were not normally distributed and clustered by year (for instance, all purchases of tenofovir were clustered by the year in which they were purchased). To benchmark prices for each ARV separately, the lowest direct manufactured cost per patient per year (LDMC) and highest direct manufactured cost per patient per year (HDMC) for 2007 was obtained. The information on the LDMC and the HDMC was obtained from Pinheiro et al [15] using the lowest and highest active pharmaceutical ingredient (API) prices reported by the WHO and the direct and indirect production costs from Brazil's public production facilities. To identify the characteristics of those countries that purchased ARVs in 2007 at the LDMC and HDMC per patient per year, two logarithmic regression models were used: one model where the dependent variable was a procurement price higher than the LDMC per patient per year and the other model where the dependent variable was a procurement price higher than the HDMC per patient per year. Independent variables were the same as used in the linear models above. ARVs were clustered by year and by type.

## Results

The majority of procurement price entries into the GPRM database are made by low-income countries. Exceptions are procurement reports of lopinavir/ritonavir and ritonavir where less than 40% of the reporting countries are low-income (Additional file 1, Tables 1 and 2).

For each ARV, countries were mainly purchasing generic products for first-line ARVs. For second-line ARVs, this was only between 8 and 66% of purchases. For first-line ARV, prices dropped between 81% and 123% in the last four years, with the exception of zidovudine (-40%) and stadivudine (0%) (Additional file 1, Table 1). Price reductions for second-line ARVs was considerably smaller, between 0% and 61%, with the exception of abacavir, which dropped by 165%. Didanosine 400 mg and ritonavir 100 mg prices were constant during the study period.

### Variables associated with price

The results of the regression model show that, generally speaking, the strongest predictor of price is whether the ARV is purchased as innovator or generic (Tables 1 and 2).

**Table 1:** Factors associated with first-line ARV prices

Antiretroviral drugs+	EFV 600 mg	3TC150 mg/ZDV 300 mg	3TC 150 mg	NVP 200 mg	d4T 40 mg	ZDC 300 mg
**HIV prevalence 2-5%**	-0.079 (-0.247, 0.089)	0.056 (-0.123, 0.236)	0.081 (-0.166, 0.328)	0.126 (-0.003, 0.256)	0.156 (-0.346, 0.035)	-0.014 (-0.179, 0.152)
**HIV prevalence >5%**	-0.120 (-0.301, 0.061)	0.140 (-0.026, 0.307)	0.143 (-0.211, 0.497)	0.100* (0.006, 0.194)	0.230 (-0.686, 0.226)	-0.008 (-0.056, 0.041)
**Lower-middle income**	0.035 (-0.088, 0.158)	0.114 (-0.088, 0.316)	0.053 (-0.242, 0.348)	0.200 (-0.208, 0.609)	0.356 (-0.277, 0.989)	0.027 (-0.212, 0.266)
**Upper-middle income**	0.027 (-0.180, 0.233)	0.356** (0.295, 0.416)	0.308* (0.160, 0.456)	0.390** (-0.295, 0.485)	0.483* (0.146, 0.820)	0.190* (0.018, 0.363)
**Volume 2**^nd^**tercile**	-0.039 (-0.264, 0.186)	-0.040 (-0.144, 0.063)	-0.041 (0.217, 0.134)	-0.149* (-0.296, -0.002)	0.120 (-0.280, 0.039)	-0.033 (-0.152 -0.085)
**Volume 3**^th^**tercile**	-0.136 (-0.407, 0.135)	-0.034 (-0.196, 0.128)	-0.045 (-0.271, 0.181)	-0.271* (-0.523, -0.020)	-0.143 (0.400, 0.113)	-0.073 (-0.226, 0.079)
API***	-0.002 (-0.015, 0.012)	-0.007* (-0.015, 0.000)	-0.013** (-0.019, -0.006)	-0.003 (-0.007, 0.001)	-0.001 (-0.013, 0.011)	0.001 (-0.011, 0.013)
**Innovator**	0.568** (0.299, 0.837)	0.736** (0.409, 1.063)	0.452* (-0.094, 0.810)	1.730** (1.366, 2.093)	1.000* (0.372, 1.629)	0.458** (0.210, 0.705)
**CHAI++**	-0.104 (-0.538, 0.331)	-0.156 (-0.356, 0.042)	-0.264* (-0.511, -0.017)	-0.105 (-0.368, 0.159)	-0.190 (-0.761, 0.382)	0.015 (-0.335, 0.365)
**PEPFAR+++**	0.007 (-0.228, 0.242)	-0.089* (-0.194, -0.015)	-0.132 (-0.491, 0.228)	-0.048 (-0.374, 0.278)	0.015 (-0.325, 0.354)	-0.069 (-0.227, 0.089)

**R-square**	0.338	0.390	0.305	0.560	0.396	0.183
**Observations**	**1514**	**1519**	**1244**	**1638**	**619**	**771**

Linear regression models whether the dependent variable is the logarithm of price. The reference categories are: HIV Prevalence <2%; Low income countries; Volume 1^st ^tercile

* significant at 5%; ** significant at 1%; Robust 95% confidence intervals in parentheses ***API = AIDS Program Effort Index; +Abbreviation of the ARV drugs: EFV = efavirenz; ZDV = zidovudine; 3TC = lamivudine; NVP = nevirapine; d4T = stadivudine; ++CHAI = Clinton Foundation HIV/AIDS initiative; +++PEPFAR = President's Emergency Plan for AIDS Relief.

Data source: Global Price Reporting Mechanism 2005 to 2008

**Table 2:** Factors associated with second-line ARV prices

Antiretroviral drugs+	ABC 300 mg	ddI 100 mg	ddI 400 mg	LPV/r 133/33 mg	RTV 100 mg	TDV 300 mg
**HIV Prevalence 2-5%**	-0.060 (-0.181, 0.061)	-0.109 (-0.298, 0.080)	-0.509** (-0.663, -0.354)	-0.405* (-0.785, -0.024)	-0.107 (-0.453, -0.239)	-0.126* (-0.222, -0.031)
**HIV Prevalence >5%**	-0.092 (-0.247, 0.063)	-0.167 (-0.335, -0.002)	-0.343** (-0.417, -0.269)	-0.629* (-1.015, -0.244)	-0.382 (-1.041, 0.277)	-0.137* (-0.265, 0.010)
**Lower-middle-income**	-0.018 (-0.155, 0.118)	0.227 (-0.417, 0.871)	0.573 (0.199, 0.948)	**0.657** (0.033, 1.282)*	-0.050 (-0.328, 0.428)	0.165 (-0.006, 0.337)
**Upper-middle-income**	-0.040 (-0.278, 0.198)	**0.407** (0.129, 0.684)*	**0.369** (0.063, 0.676)*	0.284 (-0.373, 0.942)	-0.049 (-0.313, 0.410)	0.272* (0.076, 0.467)
**Volume 2nd tercile**	-0.006 (-0.123, 0.111)	0.010 (-0.165, 0.185)	0.146 (-0.096, 0.388)	-0.039 (-0.336, 0.257)	0.017 (0.536, 0.501)	-0.117 (-0.256, 0.022)
**Volume 3th tercile**	-0.003 (-0.109, 0.103)	0.023 (-0.190, 0.235)	0.118 (-0.273, 0.509)	-0.410* (-0.637, -0.182)	-0.405* (-0.785, -0.025)	-0.158 (-0.329, 0.012)
API***	-0.004 (-0.007, 0.002)	0.008 (-0.011, 0.026)	-0.012 (-0.027, -0.004)	-0.006 (-0.018, 0.006)	0.007 (-0.013, 0.027)	0.001 (0.002, 0.004)
**Innovator**	0.662** (0.451, 0.873)	0.571** (0.411, 0.730)	0.148* (0.051, 0.245)	-0.714** (-0.989, -0.438)	-0.793 (-1.789, 0.204)	0.274** (-0.138, 0.410)
**CHAI++**	-0.143* (-0.274, -0.012)	-0.499 (-1.099, 0.102)	-0.550* (-0.982, -0.117)	-0.279* (-0.499, 0.060)	-0.666** (-1.098, -0.235)	-0.193 (-0.582, 0.197)
**PEPFAR+++**	0.054 (-0.049, 0.157)	0.046 (-0.265, 0.358)	0.347 (0.092, 0.785)	0.486* (0.091, 0.880)	-0.118* (-0.233, 0.002)	0.084 (-0.040, 0.209)

**R-square**	0.578	0.455	0.481	0.269	0.221	0.408
**Observations**	**793**	**492**	**397**	**484**	**309**	**489**

Linear regression models whether the dependent variable is the logarithm of price. The reference categories are: HIV Prevalence <2%; Low income countries; Volume 1^st ^tercile

* significant at 5%; ** significant at 1%; Robust 95% confidence intervals in parentheses; ***API = AIDS Program Index; +Abbreviation of the ARV drugs: ABC = abacavir; ddl = didanosine; LPV/r = lopinavir/ritonavir; RTV = ritonavir; TDV = tenofovir; ++CHAI = Clinton Foundation HIV/AIDS initiative; +++PEPFAR = President's Emergency Plan for AIDS Relief

Data source: Global Price Reporting Mechanism 2005 to 2008

Except lopinavir/ritonavir, the innovator is more expensive than the generic product, despite price reductions for many originator ARVs. Another relevant predictor of price of first-line ARV was a country's socioeconomic status; for lamivudine, lamivudine/zidovudine, nevirapine, stadivudine and zidovudine, upper-middle-income countries were paying statistically significant higher prices. Regarding HIV prevalence, for three second-line ARVs, the higher the countries' HIV prevalence, the lower the price. For other second-line and first-line ARVs, prevalence was not significantly associated, except nevirapine, for which countries with higher prevalence paid more.

Contrary to the commonly held assumption that countries procuring large volumes have lower prices, the data of the GPRM do not support this. Only in two out of 12 regression models, was larger volume associated with lower price (nevirapine and lopinavir/ritonavir).

Whether the country is a member of CHAI was statistically significantly associated with lower prices for three second-line drugs (abacavir, didanosine 400 mg, ritonavir) and one first-line ARV (lamivudine). In regards to the importance of being a PEPFAR priority country, the data indicate that PEPFAR priority countries were in the majority of cases not associated with the ARV price; only in the cases of didanosine 400 mg and lopinavir/ritonavir PEPFAR focus countries were paying more and the case of lamivudine/zidovudine they were paying less. Although statistically significant for two first-line ARVs, the API was not strongly influencing prices.

### Characteristics of countries paying more than minimal and maximum marginal costs for ARVs

Paying more than the LDMC and HDMC per patient per year was very strongly associated with innovator product (Table 3). Countries defined as "lower-middle-income" and "upper-middle-income" were identified as paying significantly more for ARVs than "low-income countries". Paying less than the HDMC per patient per year was associated with being member of CHAI and paying less than the LDMC per patient per year was associated with procuring very high volumes. No association was found between being a PEPFAR focus country.

**Table 3:** Factors influencing purchases higher than the lowest and the highest direct manufactured costs

	More than lowest direct manufactured costs				More than highest direct manufactured costs			
	Odds ratio	p-value	Lower 95%CI	Upper 95%CI	Odds ratio	p-value	Lower 95% CI	Upper 95% CI
**HIV prevelence < 2%**	1				1			
**HIV Prevelence 2-5%**	0.75	0.174	0.50	1.13	1.17	0.389	0.81	1.70
**HIV Prevalence >5%**	0.86	0.300	0.65	1.14	1.36	0.103	0.94	1.97
**Low income**	1				1			
**Lower-middle income**	1.43	0.079	0.96	2.14	1.63	0.010	1.13	2.37
**Upper-middle income**	4.05	0.000	2.23	7.34	2.02	0.003	1.27	3.23
PI*	0.99	0.311	0.98	1.00	0.99	0.480	0.98	1.00
**Volume 1**^st^**tercile**	1				1			
**Volume 2**^nd^**tercile**	0.76	0.211	0.50	1.17	1.28	0.060	0.99	1.65
**Volume 3**^th^**tercile**	0.56	0.016	0.35	0.90	0.69	0.055	0.48	1.01
**Innovator**	4.01	0.003	1.60	10.45	6.10	0.000	3.09	12.05
CHAI**	0.77	0.255	0.49	1.20	0.53	0.001	0.36	0.77
PEPFAR***	0.88	0.482	0.61	1.26	1.27	0.143	0.92	1.75

Pseudo R^2^	0.0994				0.1752			
Number of obs	9161				9161			

*API = AIDS Program Effort Index; **CHAI = Clinton Foundation HIV/AIDS Initiative; ***PEPFAR = President's Emergency Plan for AIDS Relief.

Data source: Global Price Reporting Mechanism 2005 to 2008

## Discussion

The results of the present study, together with findings from the literature, help to identify factors that influence prices and to make some recommendations of how procurement processes and policies could contribute to achieve better value for money.

### Generic products are very important to bring prices down

In our models, whether a country purchases an innovator product or a generic product is the strongest predictor of price. Generic prices were found to be generally lower than innovator prices, which means that, despite tiered pricing patented medications, prices have not yet caught up with the lower prices offered by generic manufacturers. As there are exceptions, a recommendation to always rely on generic products would not be beneficial. For example, the innovator product of lopinavir/ritonavir was associated with lower prices than the generic products. Countries that, due to patent law, are obligated to procure innovator products, are in a very difficult position because they have no option other than to negotiate with a monopoly provider for lower prices. Finding mechanisms to increase the negotiating power of the purchasing country may help. However, it is important to note that not all approaches to strengthen the bargaining position of purchasers have been successful. For example, data from the Andean region suggests that negotiating as a block of countries does not necessarily result in lower prices for individual countries [16].

### Country income-level

Country income-level had an important impact on the ability of a country to obtain prices lower than the LDMC or HDMC. Only in exceptional cases did countries with a higher income pay lower prices. This result is consistent with the expected result in a non-competitive market, where the manufacturers are able to sell at a price level that corresponds with the country's willingness and ability to pay. In this context, it seems important to mention that many lower- and upper-middle-income countries pay a significant proportion or all costs for ARVs compared to low-income countries often relying on donor funds.

### Procurement methods

One procurement method to influence prices is bulk procurement. Contrary to the common assumption that large volume procurement by countries results in lower prices, the results of the present study indicate that volume is in very few cases associated with lower prices which is in line with recent findings from other authors [17]. However, we found that procurement of very high volume was associated with obtaining less than the LDMC per patient per year. Some authors have found that small volumes are sometimes used to introduce a product to the country at a special low price [10]. Interestingly, on the one hand, volume gives countries more power to negotiate; on the other hand, the higher volume means that there are more people who will demand treatment and the countries are facing political pressure to respond to this need, which could reduce their negotiation power.

Another procurement method to lower prices in theory is third-party negotiation, which is used by CHAI. In our analysis, CHAI was associated with paying less than the HDMC. However, when analyzing each ARV separately, being a member of CHAI was only associated with a lower price for some ARVs. More analysis is needed to determine which countries would benefit from being a member of CHAI and how the strategies used by CHAI could be optimized to achieve further price reductions.

### Donor policies

In terms of the effect of large donors on ARV prices, being a PEPFAR focus country did not result in a lower price for all except one of the ARVs studied. It has been argued that higher prices for PEPAR countries do not necessarily mean less value for money [18]. In this study, quality was not taken into account. The ARVs that are procured by PEPFAR need to be registered with the FDA, which means that the program may choose a higher priced product over a lower-priced one, in a case in which the latter does not have a tentative approval process with the FDA [14,18]. However, in its 2008 program report, it is stated that 70% of the products from the supply chain management support of PEPFAR have the lowest international listed price (Office of the United States Global AIDS Coordinator, 2008). Interestingly, the report mentions two obstacles that make it difficult to increase the use of generics despite the objective of PEPFAR to do so: (a) the slow approval process of generics in some countries and (b) the quality concern of some buyers. It is worthwhile to investigate how many countries these barriers apply to and potential strategies to overcome them.

Another donor policy worth discussing here is the Global Fund and CHAI requirements for countries to report their pricing data. In the past decade, medicine procurement prices have been an area "that has been plagued by a troubling lack of transparency" [19]. However, this requirement of price reporting has resulted in an unprecedented accumulation of procurement price information at the global level. There has been some controversy whether increasing the transparency of prices would result in lower prices for countries. The key argument against it is that it would undermine the prices charged in higher-income countries, since the higher-income countries would demand the prices of low-income countries [6,20]. Even though it is not possible to determine how ARVs would have developed without the GPRM, the creation of the global database and the unprecedented global effort to increase price transparency nevertheless provides an important tool for more efficient procurement thorough benchmarking.

However, there is room for improvement: first, the GPRM data base is more comprehensive for low- and lower-middle-income than upper-middle-income and high-income countries, resulting in a lack of publicly available, systematically gathered information about prices for these latter countries. Particularly, upper-middle-income countries are in a double disadvantage: 1) limited price information on a global level and 2) many manufacturers do not include upper-middle-income countries in their tiered pricing system. As a result, upper-middle-income countries must negotiate individually, thus in some cases limiting their country's ability to achieve lower prices.

Second, more analysis is required on how the data is currently used by staff involved in procurement decisions and how it can be optimized to support procurement efficiency.

### Limitations

The GPRM data mainly include donor-funded procurement transactions from low-income and lower-middle-income countries, so it may not be representative of the total procurement of ARVs worldwide and results may not be generalized to all countries. Although staff of donor organizations sending the transaction data and those receiving them at WHO routinely review the reported information for entry mistakes, we checked all entries before analysis according to the procedure described above. However, we did not interview country procurement offices to verify the information reported to the donor organizations, which means that it was not possible to correct all potential errors. Taxes, tariffs and international commercial terms (INCOTERMS) are not consistently reported, so we did not include them in the analysis. Based on the US Government Accounting Office (GAO) and Management Sciences for Health (MSH), the WHO has reported that taxes, tariffs and INCOTERMS are between 3% and 15% [4]. Due to these weaknesses of the data, our results need to be interpreted in the light of the limitations of the database. As improved procurement data will be available in the near future, more analysis needs to be done to confirm our results.

This analysis did not consider pediatric formulations, only adult formulations. Other authors have analyzed the need to scale up production and increase distribution of ARV formulations that are suitable for children [21]. It is important to mention that some of the factors in our regression models are confounding; for instance, PEPFAR focus countries are mainly low-income countries. Our models do not explain most of the observed variance in the data. This suggests that a substantial part of the variation is due to factors which are not included, indicating that price determinants may be much more complex than our model suggests.

Other factors much more difficult to measure, such as corruption or the countries' willingness and capacity to negotiate with monopoly providers, could explain some differences. We used the API to account for some of these country-specific characteristics.

## Recommendations

The results presented allow identifying options of procurement processes and policies that should be considered when implementing or reforming access to ARV programs:

1. Using existing pricing data for benchmarking to improve procurement efficiency.

2. Moving away from only relying on larger volume to lower prices. It has been shown that bulk procurement alone is insufficient to lower prices.

3. Conducting more empirical analysis to identify strategies which optimize third party negotiation, particularly for CHAI.

4. PEPFAR focus countries do not have an advantage or a disadvantage over other countries to obtain lower prices. More research should focus on how to overcome quality concerns of buyers and slow registration processes of generic ARVs in affected countries as identified by PEPFAR as one of the main obstacles for the use of generics.

5. Identifying which other procurement methods result in more value for money in the future; for instance, whether strengthening negotiation skills for countries would result in lower prices.

## Competing interests

The authors declare that they have no competing interests.

## Authors' contributions

All authors were involved in the outline of the paper. VW analyzed the data and wrote the first and all following drafts of the manuscript. SF, AVM, SBA and substantially revised the drafts and re-wrote parts of the manuscript.

## Supplementary Material

Additional file 1 Click here for file
