# Supplementary material for: Factors influencing global antiretroviral procurement prices
Source: BMC Public Health. 2009 Nov 18;9(Suppl 1):S6. doi: 10.1186/1471-2458-9-S1-S6 (PMC2779508; doi:10.1186/1471-2458-9-S1-S6)
Supplement: Additional file 1 [file 1471-2458-9-S1-S6-S1.doc]

**Table 1: Median first-line ARV prices by year between 2005 and 2008 and frequency of purchases by country characteristics**

|  | **Efavirenz**  **600 mg** | **Lamivudine 150mg**  **Zidovudine**  **300 mg** | | **Lamivudine**  **150 mg** | | **Nevirapine**  **200 mg** | | **Stavudine**  **40 mg** | | **Zidovudine**  **300 mg** | |
| --- | --- | --- | --- | --- | --- | --- | --- | --- | --- | --- | --- |
| **Number of observations** | 1642 | 1732 | | 1413 | | 1685 | | 739 | | 874 | |
| **Price per patient per year**  Median (price at 25% and 75% of the sample) | | | | | | | | | | | |
| **2005** | 350.4  (346.8 - 397.9) | | 211.7  (175.2 - 240.9) | | 73.0  (65.7 - 80.3) | | 87.6  (73.0 – 233.6) | | 51.1  (43.8 - 58.4) | | 153.3  (131.4 – 182.5) |
| **2006** | 244.6  (244.6 – 284.7) | | 145  (138.7 – 175.2) | | 58.4  (51.1 - 65.7) | | 65.7  (58.4 - 65.7) | | 36.5  (36.5 - 51.1) | | 146  (131.4 - 146) |
| **2007** | 193.5  (149.7 – 237.3) | | 124.1  (109.5 -146.0) | | 43.8  (36.5 – 58.4) | | 43.8  (43.8 - 51.1) | | 29.2  (21.9 – 36.5) | | 109.5  (102.2 - 138.7) |
| **2008** | 157.0  (142.4 - 180.7) | | 116.8  (116.8 – 135.1) | | 36.5  (36.5 – 58.4) | | 43.8  (36.5 - 51.1) | | 51.1  (21.9 – 65.7) | | 109.5  (102.2 - 116.8) |
| **Price variation between 2005 and 2008** | -123.2% | | -81.3% | | -100% | | -100% | | 0% | | -40.0% |
| **HIV prevalence <2%** | 49.8 | | 50.6 | | 42.1 | | 50.8 | | 45.9 | | 41.9 |
| **HIV prevalence 2-5%** | 13.7 | | 16.1 | | 15.6 | | 13.2 | | 13.5 | | 17.4 |
| **HIV prevalence >5%** | 36.5 | | 33.4 | | 42.3 | | 35.9 | | 40.6 | | 40.7 |
| **% Low- income** | 64.2 | | 62.5 | | 48.4 | | 63.7 | | 46.8 | | 57.0 |
| **% Lower- middle- income** | 19.3 | | 24.8 | | 25.6 | | 20.9 | | 24.8 | | 22.8 |
| **% Upper- middle- income** | 14.9 | | 11.0 | | 23.8 | | 14.1 | | 26.8 | | 17.3 |
| **% High- income*** | 1.6 | | 1.6 | | 2.1 | | 1.3 | | 1.6 | | 3.0 |
| **% Least Developed Countries** | 55.6 | | 56.2 | | 41.3 | | 55.9 | | 42.0 | | 47.8 |
| **Number of units per purchase**  Median (at 25 % and 75% of the sample) | 50,715  (6,600 - 227,700) | | 104,910  (15,180 - 390,000) | | 88,020  (16,860 - 345,300) | | 49,920  (8,040 - 281,280) | | 36,000  (5,760 - 113,580) | | 25,110  (7,200 - 96,000) |
| **% Generic** | 85.6 | | 84.8 | | 85.1 | | 86.4 | | 85.1 | | 85.6 |
| **API*** mean±sd | 61.6±9.6 | | 61.1±10.1 | | 62.7±10.2 | | 61.0.±9.9 | | 61.0±11.1 | | 62.6±10.1 |
| **% Clinton Foundation HIV/AIDS Initiative** | 88.9 | | 89.1 | | 89.1 | | 90.0 | | 90.4 | | 88.1 |
| **% PEPFAR**** | 37.0 | | 34.4 | | 43.7 | | 35.3 | | 41.4 | | 41.2 |

Table legend: *API= AIDS Program Effort Index; PEPFAR= The United States President’s Emergency Plan for AIDS Relief

Robust 95% confidence intervals in parentheses; * significant at 5%; ** significant at 1%;

Data source: Global Price Reporting Mechanism 2005 to 2008

**Table 2: Median second-line ARV prices by year between 2005 and 2008 and frequency of purchase by country characteristics**

|  | **Abacavir**  **300 mg** | **Didanosine**  **100 mg** | **Didanosine**  **400 mg** | **Lopinavir**  **133 mg**  **Ritonavir**  **33 mg** | **Ritonavir**  **100 mg** | **Tenofovir**  **300 mg** |
| --- | --- | --- | --- | --- | --- | --- |
| **Number of observations** | 844 | 537 | 423 | 551 | 332 | 504 |
| **Price per patient per year**  Median price (price at 25% and 75% of the sample) | | | | | | |
| **2005** | 890.6  (890.6 - 956.3) | 306.6  (306.6 – 401.5) | 288.4  (288.4 -1120.6) | 613.2  (503.7-3285.0) | 87.6  (73.0 – 102.2) | 299.3  (211.7 -324.9) |
| **2006** | 635.1  (540.2 - 894.3) | 277.4  (233.6 – 335.8) | 288.4  (277.4 – 507.4) | 591.2  (525.6 – 2124.3) | 87.6  (80.3 – 485.5) | 208.1  (208.05 - 266.5) |
| **2007** | 401.5  (372.3 – 467.2) | 306.6  (248.2 - 350.4) | 288.4  (284.7 – 361.4) | 1007.4  (591.3 – 1554.9) | 87.6  (80.3 – 532.9) | 208.1  (167.9 – 222.7) |
| **2008** | 335.8  (313.9 – 401.5) | 248.2  (189.8 – 292.0) | 288.4  (284.7 – 288.4) | 503.7  (438.0 – 580.4) | 87.6  (80.3 – 138.7) | 186.2  (153.3 - 208.0) |
| **Price reduction** | -165.2% | -23.5% | 0% | -21.7% | 0% | -60.7% |
| **HIV prevalence <2%** | 40.3 | 34.3 | 52.7 | 46.5 | 40.9 | 40.9 |
| **HIV prevalence 2-5%** | 19.1 | 10.2 | 13.7 | 8.4 | 16.9 | 16.9 |
| **HIV prevalence >5%** | 40.6 | 55.5 | 29.1 | 45.2 | 42,4 | 42.2 |
| **% Low-income** | 62.1 | 40.2 | 65.5 | 32.3 | 35.8 | 63.7 |
| **% Lower- middle-income** | 22.0 | 21.2 | 21.3 | 27.2 | 28.0 | 26.0 |
| **% Upper- middle-income** | 14.2 | 37.1 | 9.2 | 38.1 | 35.0 | 8.7 |
| **% High-income** | 1.7 | 1.5 | 4.02 | 2.4 | 1.2 | 1.6 |
| **% Least Developed Countries** | 48.3 | 34.1 | 57.0 | 25.4 | 30.7 | 47.0 |
| **Number of units per purchase**  Median (at 25% and 75% of the sample) | 18,000  (3,180 - 60,000) | 13,200  (3,360 - 47,280) | 12,780  (2,400 - 30,000) | 33,600  (13,200 - 100,800) | 16,800  (7,248 - 46,200) | 21,540  (4,785 - 76,050) |
| **% Generic** | 65.9 | 49.7 | 8.0 | 8.0 | 9.0 | 55.2 |
| **API** mean±sd | 63.0±9.9 | 65.5±10.1 | 61.5±10.0 | 62.8±11.3 | 63.9±10.4 | 64.2±9.8 |
| **% Clinton foundation** HIV/AIDS initiative | 84.1 | 91.4 | 77.0 | 81.0 | 77.2 | 79.3 |
| **% PEPFAR** | 45.6 | 54.2 | 32.9 | 40.5 | 46.1 | 48.8 |

Table legend: *API= AIDS Program Effort Index; CHAI=Clinton Foundation HIV/AIDS initiative; PEPFAR=President’s Emergency Plan for AIDS Relief; Robust 95% confidence intervals in parentheses; * significant at 5%; ** significant at 1%;

Data source: Global Price Reporting Mechanism 2005 to 2008
